# The scientific study of inspiration in the creative process: challenges and opportunities

**DOI:** 10.3389/fnhum.2014.00436

**Published:** 2014-06-25

**Authors:** Victoria C. Oleynick, Todd M. Thrash, Michael C. LeFew, Emil G. Moldovan, Paul D. Kieffaber

**Affiliations:** Department of Psychology, College of William and MaryWilliamsburg, VA, USA

**Keywords:** inspiration, creativity, insight, effort, approach motivation

## Abstract

Inspiration is a motivational state that compels individuals to bring ideas into fruition. Creators have long argued that inspiration is important to the creative process, but until recently, scientists have not investigated this claim. In this article, we review challenges to the study of creative inspiration, as well as solutions to these challenges afforded by theoretical and empirical work on inspiration over the past decade. First, we discuss the problem of definitional ambiguity, which has been addressed through an integrative process of construct conceptualization. Second, we discuss the challenge of how to operationalize inspiration. This challenge has been overcome by the development and validation of the Inspiration Scale (IS), which may be used to assess trait or state inspiration. Third, we address ambiguity regarding how inspiration differs from related concepts (creativity, insight, positive affect) by discussing discriminant validity. Next, we discuss the preconception that inspiration is less important than “perspiration” (effort), and we review empirical evidence that inspiration and effort both play important—but different—roles in the creative process. Finally, with many challenges overcome, we argue that the foundation is now set for a new generation of research focused on neural underpinnings. We discuss potential challenges to and opportunities for the neuroscientific study of inspiration. A better understanding of the biological basis of inspiration will illuminate the process through which creative ideas “fire the soul,” such that individuals are compelled to transform ideas into products and solutions that may benefit society.

## Introduction

Describing his creative process, Mozart observed, “Those ideas that please me I retain in memory, and am accustomed, as I have been told, to hum them to myself. If I continue in this way,” he writes, “it soon occurs to me how I may turn this or that morsel to account so as to make a good dish of it… All this fires my soul” (Harding, 1948). Mozart’s depiction of inspiration possesses all of the core elements of the modern scientific inspiration construct—appreciation of new or better possibilities (“ideas that please me”), passive evocation (“it…occurs to me”), and motivation to bring the new possibilities into fruition (turning a morsel into a dish; “fires my soul”). Like Mozart, writers, artists, and other creators commonly emphasize the importance of inspiration in the creative process (Harding, 1948). Despite this, until recently, scientists have given little attention to inspiration.

Perhaps it is not surprising that inspiration has received little attention within the scientific community, given the numerous challenges that the inspiration concept has presented. Among these challenges have been (a) a lack of clarity about the meaning of inspiration; (b) difficulty of operationalization; (c) ambiguity about whether inspiration is distinct from related constructs; (d) preconceptions that inspiration is unimportant relative to “perspiration,” and (e) a variety of barriers to neuroscientific investigation. The overarching goal of this article is to address each of these challenges and to point to opportunities for expanding upon the emerging scientific literature on inspiration. We address the first challenge, ambiguity of definition, in the next section.

## Conceptualization

The term “inspiration” has been used in a variety disciplines (e.g., literary criticism, theology, psychology) and literatures within psychology (e.g., social comparison, humanism, creative process; for a review, see Thrash and Elliot, 2003). Often the term is not defined, is used interchangeably with other constructs, or is referenced only to be critiqued as mythical, unimportant, or unscientific. Further complicating matters, inspiration historically has been studied in a domain-specific manner, with little communication between researchers across domains. Recognizing the need for a unified, integrated definition of the inspiration construct, Thrash and Elliot (2003, 2004) undertook the task of developing a domain-general conceptualization that drew upon the core commonalities across diverse literatures. These efforts have yielded three complementary frameworks for conceptualizing inspiration that focus on different aspects of construct definition: core characteristics, component processes, and the transmission model. In this section, we review these domain-general conceptualizations and then show how they may be applied specifically to the case of inspiration to create.

### Tripartite conceptualization

The *tripartite conceptualization* (Thrash and Elliot, 2003) specifies the three core characteristics of the state of inspiration: *evocation*, *transcendence*, and *approach motivation*. Evocation refers to the fact that inspiration is *evoked* rather than initiated volitionally by the individual. In other words, one does not feel directly responsible for becoming inspired; rather, a stimulus object, such as a person, an idea, or a work of art, evokes and sustains the inspiration episode. During an episode of inspiration, the individual gains awareness of new possibilities that *transcend* ordinary or mundane concerns. The new awareness is vivid and concrete, and it surpasses the ordinary constraints of willfully generated ideas. Once inspired, the individual experiences a compelling *approach* *motivation* to transmit, actualize, or express the new vision. This set of three characteristics is intended to be minimally sufficient to distinguish the state of inspiration from other states.

### Component processes

Inspiration may be conceptualized not only in terms of the characteristics of the inspired state, but also in terms of the temporally and functionally distinct processes that compose an episode of inspiration. Thrash and Elliot (2004) argued that inspiration involves two distinct processes—a relatively passive process that they called being inspired *by*, and a relatively active process that they called being inspired *to*. The process of being inspired *by* involves appreciation of the perceived intrinsic value of a stimulus object, whereas the process of being inspired *to* involves motivation to actualize or extend the valued qualities to a new object. For example, one might be inspired by a breathtaking sunrise, or by the elegance of a new idea that arrives during an insight or “aha” moment. Thereafter one might be inspired to paint or undertake a new research project. The individual can, at any time, look to (or recall) the evoking stimulus for motivational sustenance. Thrash and Elliot (2004) further proposed that the process of being inspired *by* gives rise to the core characteristics of evocation and transcendence, whereas the process of being inspired *to* gives rise to the core characteristic of approach motivation.

These component processes are posited to be present across diverse manifestations of inspiration. Thrash and Elliot (2004) asked participants to produce narratives recalling either a time when they were inspired or a baseline experience (control condition). The inspiration narratives spanned topics such as becoming animated by a scientific or artistic insight, discovering one’s calling, being influenced by a role model to succeed or live virtuously, and realizing that greatness is possible in response to an unexpected success. Despite superficial differences in narrative content, the inspiration narratives shared the underlying themes of having one’s eyes opened during an encounter with a person, object, event, or idea (i.e., being inspired “by”), and wishing to express or actualize one’s new vision (i.e., being inspired “to”).

### Transmission model

From a less descriptive and more theoretical standpoint, inspiration may be conceptualized in terms of its purpose or function (Thrash and Elliot, 2004; Thrash et al., 2010b). Whereas simpler forms of approach motivation serve the function of movement toward and attainment of desired goal objects (e.g., food or affiliation), inspiration is posited to serve a unique approach function: it motivates the transmission or expression of the newly appreciated qualities of the evoking object (Thrash and Elliot, 2004; Thrash et al., 2010b). Inspiration thus serves the role of a mediator in a statistical sense. For instance, certain virtues that one observes in another person may lead to inspiration, which, in turn, leads the inspired individual to pursue these same virtues in a future self. Similarly, a creative seminal idea may inspire the individual, compelling him or her to bring the idea into fruition in the form of a creative invention, poem, or other tangible product.

### Inspiration to create

The general inspiration construct as conceptualized above may be applied straightforwardly to the specific domain of creative activity. From the perspective of the tripartite conceptualization, the general characteristic of transcendence takes the form of *creativity—*the new or better possibilities are appreciated specifically for their creative potential. Regarding the component process conceptualization, the process of being inspired *by* is prompted by the emergence of creative ideas in consciousness, often during a moment of insight. Under optimal conditions (e.g., if the idea is actionable, and the person has the capacity for approach motivation), the process of being inspired *by* gives way to the process of being inspired *to*, which motivates action. Regarding the transmission model, creative inspiration often takes a specific form of transmission called *actualization* (Thrash et al., 2010b), in which one is inspired to bring a creative idea into fruition (i.e., the desirable features of the elicitor are transmitted from a seminal idea to a completed product).

We emphasize that, according to our conceptualization, inspiration is not posited to be the *source* of creative ideas. Instead, inspiration is a motivational *response* to creative ideas. Thus inspiration explains the transmission, not the origin, of creativity. This distinction is critical for at least three reasons. First, claiming that creativity comes from inspiration would not aid scientific understanding, much as attributing creativity to a “muse” would be an exercise in labeling a mysterious cause, not a scientific explanation. Second, scientists have already developed a variety of scientific constructs and theories to explain the origins of creative ideas, which include situational, dispositional, self-regulatory, cognitive, historical, and neurological processes (e.g., Koestler, 1964; Rothenberg, 1979; Martindale, 1990; Finke et al., 1992; Sternberg and Davidson, 1995; Amabile, 1996; Feist, 1998; Bowden and Jung-Beeman, 2003; Simonton, 2003; Baas et al., 2013). In contrast, scientists have given relatively little attention to the processes through which creative ideas are transformed into creative products. The inspiration construct helps fill this gap in the research literature. Finally, because this conceptualization of creative inspiration is derived from a general conceptualization, it is consistent with usage of the inspiration construct in other literatures. For instance, creative inspiration is a response to (not the cause of) creative ideas, much as interpersonal inspiration is a response to (not the cause of) virtuous qualities in others.

## Operationalization

Given the personal nature and elusiveness of the experience of inspiration, how can it possibly be measured in the laboratory? One might be tempted to throw up one’s hands and turn instead to something that is more amenable to direct experimental control.

### The value of self-report

We maintain that self-report is a straightforward and appropriate method for operationalizing inspiration, because the inspiration construct is inextricably intertwined with a distinctive phenomenological experience. Numerous creators have claimed—through conscious self-reports—that they experience inspiration and that this experience is critical to their creative process (Harding, 1948). Operationalizing inspiration through self-report allows researchers to put such claims to the test.

Thrash and Elliot (2003) developed a trait measure of inspiration called the Inspiration Scale (IS). Although the term “trait” has a variety of connotations, trait inspiration refers to nothing other than individual differences in the tendency to experience the state of inspiration. Because inspiration is a construct that is meaningful in individuals’ lives but underappreciated by psychologists, the measure was designed to be straightforward and face valid. Items include statements such as, “Something I encounter or experience inspires me” and “I am inspired to do something.” The IS has two internally consistent 4-item subscales: inspiration frequency and intensity. Both subscales are internally consistent, with Cronbach’s αs equal to or greater than 0.90. The two subscales have been demonstrated to be highly correlated (*r* = 0.60 to 0.80), and therefore scores may be summed to form an internally consistent 8-item index of overall inspiration. The IS demonstrates measurement invariance across time (2 months) and across populations (patent holders, university alumni), indicating that the underlying latent constructs have comparable meaning at different points in time and in different populations. Two-month test-retest reliabilities for both subscales are high, *r* = 0.77. In short, the IS has excellent psychometric properties. Notably, the intensity subscale has been adapted for use as a state measure (e.g., Thrash and Elliot, 2004; Thrash et al., 2010a).

Some may worry that self-reported inspiration cannot be trusted, that it is not objective, or that it does not provide a full explanation. We respond to each of these potential limitations. First, inspiration, as assessed with the IS, tends to be unrelated or weakly related to social desirability, and its predictive validity is robust when social desirability is controlled^1^ (Thrash and Elliot, 2003; Thrash et al., 2010a). Second, although the IS provides a subjective indicator of inspiration, scores on this measure have been linked to a variety of external criteria and objective outcomes, as reviewed in the following section. Moreover, consciousness plays a critical role in the simulation of future action in humans (Baumeister and Masicampo, 2010) and may be necessary for inspired action. Accordingly, conscious self-report is intrinsically appropriate to the construct. Finally, we recognize that self-report measures may leave some researchers with a hunger for lower-level explanations, such as those involving physiological or neurological processes, but we see this as an opportunity rather than a problem—the inspiration construct may see an exciting second generation of research regarding neural underpinnings. In this case, self-reported inspiration provides a “bootstrap” that may guide researchers to underlying process. Although it is true that the self-report method is limited in some ways, it offers a well-validated starting point for neuroscientific investigations. Moreover, not investigating inspiration on the grounds that it is measured by self-report would lead researchers to overlook a critical predictor of creative output, the biological underpinnings of which would remain undiscovered.

### The place of inspiration in creativity research paradigms

The field of creativity assessment is active and dynamic, and thus a review of the literature is well beyond the scope of this article (for a review, see Plucker and Makel, 2010). We note, however, that the dominant research paradigms used in the study of creativity have unwittingly precluded attention to inspiration. Creativity is most often assessed using tests of creative ideation (e.g., Alternate Uses) or creative insight (e.g., Remote Associates Test). While such tests are very practical in laboratory contexts and allow researchers to focus on the processes underlying the emergence of creative ideas, they do not allow participants to transform creative ideas into creative products. Failure to accommodate the idea actualization process—that is, creation *per se—*renders inspiration speciously immaterial to the creative process. If the function of inspiration within the context of creativity is the actualization of creative ideas into creative products, useful paradigms must allow for idea actualization. Product-based assessments, such as the Consensual Assessment Technique (CAT; Amabile, 1982) and analysis of patent data, are the gold standard if one wishes to investigate the unique contribution of inspiration to the creative process.^2^ In fact, relevance to inspiration aside, assessment of creative products is considered by some to be the most appropriate and valid operationalization of creativity (Baer et al., 2004; Baer and McKool, 2009).

## Discriminant validity

Ambiguity about whether inspiration is distinct from other constructs has been another impediment to research activity. If one presumes that inspiration is the same thing as, for example, creativity or insight, then one has no reason to study it. In this section, we clarify the distinctions between inspiration and several other constructs (creativity, insight, and positive affect).

### Inspiration and creativity

While there is considerable variability in the definition and usage of the term creativity within psychology (Silvia and Kaufman, 2010), there is some degree of consensus that creativity implies two qualities: novelty and usefulness (e.g., Feist, 1998; Plucker et al., 2004). We find it useful to explicitly conceptualize creativity as an *appraisal* of novelty and usefulness that may be applied to any of a variety of objects, particularly ideas and resulting products. Depending on the aims of the research, this appraisal may be made by the creator herself, by gatekeepers within a field, by an audience, or through various other operationalizations available to the researcher. We note that researchers often appear to have either ideas or products in mind as the ultimate objects of creativity appraisals, even when the term “creative” precedes other nouns (e.g., creative activity (Simonton, 2000), creative insights (Csikszentmihalyi and Sawyer, 1995), creative personalities (Feist, 2010), creative states (Jamison, 1989), or creative processes (Kris, 1952)).

Although the terms inspiration and creativity have occasionally been used synonymously (e.g., Schuler, 1994; Chamorro-Premuzic, 2006), our conceptualizations of inspiration and creativity involve a clear delineation. Creativity is an appraisal of novelty and usefulness that may apply (to various degrees) to content at any point in the creative process, from a seminal idea to the completed product. Inspiration, in contrast, is a motivational state. We posit that inspiration is often elicited when a creator appraises his or her idea as creative, and it is posited to motivate actualization of the idea in the form of a product that is likewise appraised (by its creator and perhaps others) as creative. We discuss empirical support for these proposals below.

### Inspiration and insight

Conflation of inspiration with insight is common in everyday language.^3^ An individual might exclaim, “I had an inspiration,” where “inspiration” refers to the idea itself, not to the motivational response. In the scientific context, the term insight has been used to describe the process by which a problem solver suddenly moves from a state of not knowing how to solve a problem to a state of knowing how to solve it (Mayer, 1992). Within the creativity context, insight has also been conceptualized as the cognitive content that enters consciousness suddenly; the “aha!” moment (Csikszentmihalyi and Sawyer, 1995). Regardless of its exact usage, insight can be differentiated from inspiration in terms of its theoretical function. Whereas insight research is an attempt to explain the cognitive mechanisms, such as restructuring (Ohlsson, 1984), by which ideas enter awareness, inspiration research is an attempt to explain the motivational response that often (but not always) follows creative insight (see Thrash et al., 2010b).

If inspiration always followed from insight, then perhaps the inspiration construct would be superfluous. However, inspiration does not always follow. Thrash et al. (2010b) found that creative ideation tends to lead to inspiration but that this effect is moderated by individuals’ approach temperament (i.e., sensitivity to reward; Elliot and Thrash, 2010). Individuals with a strong approach temperament tend to get inspired to create in response to creative insight, whereas individuals with a weak approach temperament report feeling a lack of inspiration in spite of their insight. Inspiration thus has important implications for the behavioral transmission of a creative insight into a creative product.

Recent work on the phenomenology of insight offers hints about how insight may lead to inspiration. Abrupt changes in processing fluency during insight have been found to endow an individual with elevated levels of positive affect (PA) and perceived truth regarding his or her solution (Topolinski and Reber, 2010). Given that PA is involved in both the insight “aha” experience and inspiration, it may facilitate a fluid transition from insight to inspiration. Moreover, perceiving one’s solution as true, a consequence of insight, may bolster inspired motivation. As we have noted, however, insight can occur without inspiration. Dispositional factors of the individual (e.g., low approach temperament) and situational factors (e.g., contexts in which opportunities for transmission are not available) can impede inspiration. Likewise, inspiration can occur outside of the problem-solving context and without a discrete and sudden insight.

### Inspiration and positive affect

Activated PA, a high-arousal form of pleasant affect, is the strongest known correlate of inspiration (Thrash and Elliot, 2003). Indeed, the term “inspired” appears on the PANAS measure of activated PA (Watson et al., 1988). Because activated PA is often present during states of approach motivation (Watson et al., 1999), it particularly resembles the inspired *to* component process.

Although inspiration and activated PA overlap to some degree empirically and conceptually, considerable evidence supports their discriminant validity. First, inspiration and activated PA are factorially distinct (Thrash and Elliot, 2003). Second, consistent with the tripartite conceptualization of inspiration, experiences of inspiration involve greater levels of transcendence and lower levels of volitional control and ascriptions of personal responsibility (indicative of “evocation”) compared to experiences of activated PA (Thrash and Elliot, 2004). Third, inspiration and activated PA have different proximal and distal antecedents (Thrash and Elliot, 2004). Activated PA is triggered proximally by reward salience (environmental cues and perceptions that something desired is attainable) and distally by approach temperament. In contrast, inspiration is triggered proximally by experiences of insight and distally by openness to experience. Finally, inspiration and activated PA have different distributions across days of the week; on Fridays, for instance, activated PA is at its peak while inspiration is at its trough (Thrash, 2007).

## Inspiration, perspiration, and creativity

Perhaps the most pernicious obstacle to research on inspiration has been the longstanding belief that it is perspiration, and not inspiration, that is critical for creative output. Thomas Edison, regarding his work, once remarked that, “what it boils down to is one per cent inspiration and ninety-nine per cent perspiration” (Edison, 1903). This comment has sometimes been offered in support of the idea that effort is important to creativity and that inspiration, by comparison, is unimportant (e.g., Martindale, 1989, 2001; Sawyer, 2006). Furthering this line of reasoning, Fehrman and Petherick (1980) offered an account of why inspiration nonetheless endures as a folk explanation of creativity: when individuals are exposed to creative works, they misattribute creators’ effort to inspiration, unaware how much effort was required to produce the work. It appears that reasoning such as this has precluded attention to a legitimate role of inspiration in the creative process.

Empirical data related to inspiration, perspiration, and creativity are now available for consideration. A number of studies indicates that inspiration is a robust predictor of creativity. At the between-person (i.e., trait) level, inspiration and creative self-concept are positively correlated, and inspiration predicts longitudinal increases in creative self-concept (Thrash and Elliot, 2003). Trait inspiration also predicts objective indicators of creative output. In a sample of U.S. patent holders, inspiration frequency was found to predict the number of patents held (Thrash and Elliot, 2003). Inspiration also predicts creativity at the within-person level, such that inspiration and self-reported creativity fluctuate together across days (Thrash and Elliot, 2003).

In three studies of different types of writing (poetry, science, and fiction), self-reported state inspiration during the writing process uniquely predicted creativity of the final product, as assessed by expert judges using the CAT (Thrash et al., 2010b). These findings held when a variety of covariates (e.g., openness to experience, effort, activated PA, awe) were controlled. Finally, inspiration has been shown to mediate between the creativity of seminal ideas and the creativity of final products in a manner consistent with the posited transmission function^4^ of inspiration (Thrash et al., 2010b). Covariates of inspiration (effort, activated PA, awe) failed to mediate transmission, indicating that the transmission function is unique to inspiration.

Having established a relation between inspiration and creativity, we now consider the role of “perspiration” in the creative process. Notably, Thrash et al. (2010b) documented a positive relation, rather than a negative relation, between inspiration and effort, indicating that these constructs are not mutually exclusive as the Edison quote may imply. The assumption that the presence of effort indicates low levels of inspiration is further challenged by a positive relation between inspiration and the work-mastery component of need for achievement (Thrash and Elliot, 2003). Both of these findings were documented at two statistically independent levels of analysis (between-persons, within-persons).

Certainly effort is important to the creative process, but its role is different than that of inspiration. Whereas writers’ inspiration predicts the creativity of the product, writers’ effort predicts the technical merit of the product (Thrash et al., 2010b). Thus inspiration and effort are unique predictors of different aspects of product quality. Moreover, screen capture data indicate that inspiration is involved in the automatic/generative aspects of the writing process (e.g., inspired writers produce more words and retain more of their original typing), whereas effort is related to controlled self-regulation (e.g., writers who exert effort delete more words and pause more to think; Thrash et al., 2010b). In short, inspiration and “perspiration” are not mutually exclusive, and they contribute in qualitatively different ways to the creative process and product.

The question of whether the audience correctly infers the presence of inspiration remains. The misattribution hypothesis states that it is the creator’s *effort* that predicts the creativity of the product but that the audience incorrectly attributes this creativity to *inspiration* in the creator. An alternative to this model is the possibility that the audience correctly infers inspiration (Bowra, 1977). Thrash et al. (2010b) tested these competing hypotheses. Readers were found to correctly attribute creativity to writers’ inspiration; likewise, they correctly attributed technical merit to writers’ effort. These results, in addition to providing the first empirical evidence that readers can make veridical inferences about writers’ motivational states, indicate that folk notions of the importance of inspiration are borne out by empirical data.

The psychological science of inspiration, as well as its relation to creativity, is now well-established. Inspiration has been conceptualized through integration of usages in diverse literatures, operationalized using a well-validated measure, discriminated from related constructs, and linked to creativity in multiple populations, contexts, and levels of analysis. Prior work provides a solid foundation on which investigations into the neuroscience of inspiration can rest.

## Inspiration in the neuroscience laboratory

In most respects, the challenges associated with studying creative inspiration are similar regardless of whether one approaches the topic as a neuroscientist, a psychologist, etc. Therefore, the preceding general challenges and solutions are also relevant specifically in the neuroscience context. However, we reiterate the importance of attending carefully to construct definition, because the term “inspiration” has occasionally been used in the neuroscience literature to refer to constructs that are quite different than the inspiration construct that we have discussed. In their classic EEG studies of the creative process, for instance, Martindale and Hasenfus (1978) used the terms inspiration and elaboration to refer to the stages that precede and follow, respectively, creative insight (see Kris, 1952, for a precedent for such usage in psychoanalysis). Inspiration as we have defined it—i.e., as a conscious motivational state rather than as a stage—is more likely to occur during Martindale and Hasenfus’s elaboration stage than during the inspiration stage. We now turn to challenges that are particularly relevant within a neuroscience context.

One obstacle in studying inspiration in the laboratory is the impossibility of direct manipulation through exposure to exogenous elicitors. If one seeks to elicit inspiration through use of some kind of “inspiring” stimulus, then the manipulated elicitor is the independent variable and inspiration is a dependent variable. Thus caution is needed regarding causal inference, despite use of the experimental method (Thrash et al., 2010a). Although inspiration cannot be directly manipulated through exposure to exogenous stimuli, a researcher may build a case for causality using manipulation of elicitors in combination with statistical controls and cross-lagged analyses, as demonstrated by Thrash et al. (2010a). We note that these problems are not unique to the study of inspiration. Emotions, insight, and many other constructs elude strict experimental control; at best, they may be “elicited” rather than “manipulated”.

A related challenge is that it may be difficult to capture authentic or intense experiences of inspiration in a laboratory setting, given that inspiration is elusive for certain individuals or under certain circumstances. One solution may be to, in effect, lower the threshold for what constitutes an episode of inspiration. Thrash and Elliot (2004), for instance, studied “daily inspiration” using experience sampling methods, and we suggest that such tolerance for less intense manifestations of inspiration can be extended to a laboratory study. Much as creativity is not the same thing as genius (Bruner, 1962), inspiration is a matter of degree, and moderate levels might be achievable even in some invasive neuroscience paradigms.

A third challenge is the need for repeatable trials and time-locking. Brain imaging techniques (e.g., fMRI, EEG, MEG) require designs in which the mental event under consideration may be (a) temporally isolated so that the recorded data and the mental event can be time-locked to an eliciting stimulus and (b) elicited repeatedly during a recording session in order to improve the signal-to-noise ratio (Dickter and Kieffaber, 2013). One possible method to address these requirements is to use participant self-report (indicating the onset of inspiration) as the time-locking event. Suppose, for example, participants invent captions for each of a series of photographs (a highly-repeatable activity) and report on levels of inspiration at the moment of getting an idea for each caption. Bowden and Jung-Beeman (2007) used a method similar to this in order to identify processes that distinguish solutions involving the experience of insight from those that do not. We caution, however, that inspiration generally is more prolonged in time than is insight (particularly when considerable activity is needed to actualize an idea), and therefore methods that capture subsequent variability in inspiration across time—not just the level of inspiration at the moment of insight—will be particularly valuable.

One such method for capturing variability in inspiration across time, while simultaneously reducing the burden of eliciting inspiration repeatedly, is to record electrical brain activity using a non-invasive technique (such as EEG) during the creative process. For instance, if researchers record screen capture data during the writing process as in Thrash et al. (2010b), they can subsequently play back the recording to participants and collect continuous measures of recalled inspiration during the creative process (e.g., using a dial or slider input device). These ebbs and flows of inspiration can then be linked to variability in neural processes.

The difficulties associated with eliciting inspiration in order to study it at the within-person level may also be addressed by simply focusing on the individuals who are likely to be inspired (i.e., those who are high in trait inspiration). Elicitation may be circumvented altogether by examining structural brain differences between groups known to be high versus low in trait inspiration. One may separate groups into “more inspired” and “less inspired” using the IS. Additionally, as individuals higher in trait inspiration tend to exhibit greater levels of openness and extraversion, one might expect, for example, reduced latent inhibition and increased activity in the ventral tegmental area dopamine projections (Ashby et al., 1999; Depue and Collins, 1999; Peterson et al., 2002) for these individuals. Thus, inspiration’s nomological network can serve as an informative starting point for between-person neurological analyses.

Next, we consider the question of where to look in the nervous system. While at present there is no neuroscience of the inspiration construct *per se*, literatures on related constructs can offer us some hints.

Insight relates to inspiration within the tripartite conceptualization in terms of both evocation and transcendence, and within the component processes model as the initial event that often leads one to become inspired *by*. During “Aha!” moments, one *transcends* a mental set and experiences a conceptual expansion (Abraham et al., 2012), and the experience feels automatic and unexpected; it feels *evoked* (Bowden et al., 2005). Therefore, certain neural components involved in insight experiences may be present at the onset of an inspiration episode. However, given that the literature on the neural correlates of insight is complex and that neural processes are under debate (Dietrich and Kanso, 2010), we caution against relying too heavily upon any one finding in guiding work on inspiration.

As inspiration involves not only transcendence and evocation, but also approach motivation, we may also look to the neuroscience literature on states of approach motivation (Elliot, 2008). There exists a burgeoning literature on approach motivation and appetitive affect, with attention to underlying neuronal circuitry (e.g., Bradley et al., 2001; Aron et al., 2005; Junghöfer et al., 2010), subcortical reward systems (e.g., Rosenkranz and Grace, 2002; Wise, 2004; Alcaro et al., 2007), neurotransmitters (e.g., Bassareo et al., 2002; Hoebel et al., 2008), and neurohormones (e.g., Frye and Lacey, 2001; Frye and Seliga, 2003; Frye, 2007). Findings in this area may offer suggestions for the neural underpinnings of the inspired *to* process.

Although the neurological findings regarding certain aspects of the inspiration construct can offer clues, the neural components of these pieces alone are unlikely to tell the full story. After all, we have already argued above that inspiration is not the same thing as insight or activated PA, nor is it the sum of these parts. For instance, an individual could be in an appetitive motivational state at the same time that he or she gets a creative insight, but he or she would not be inspired if the appetitive state reflects anticipation of eating, rather than of bringing the idea into fruition. The evoking object, in this case, the insight, does not meaningfully relate to the motivational object. The critical question for neuroscience is how processes related to generation of creative ideas recruit appetitive motivational processes, such that individuals respond to creative ideas not with indifference, but rather with a feeling of being compelled to act. How exactly does the prospect of turning a morsel into a dish fire the soul, as Mozart put it (in the opening quotation)?

In the initial stages of research on the neurological basis of inspiration, it may be useful to begin with a focus on overall inspiration instead of particular aspects or component processes. Inspiration as a unified concept can be measured quite efficiently using the 4-item intensity subscale of the IS (Thrash and Elliot, 2004). If necessary, inspiration could be assessed with a single item from the IS. Such items are surprisingly effective at capturing the full inspiration construct as we have defined it (Thrash et al., 2010b).

## Conclusion

Writers, artists, and other creators have long argued that inspiration is a key motivator of creativity. Over the past decade, scientists have tested and found strong support for these claims. Scientific progress has required overcoming a number of challenges, including definitional ambiguity, difficulties of operationalization, ambiguities about discriminant validity, and skepticism about the importance of inspiration relative to perspiration. By developing an integrative conceptualization, operationalizing inspiration with the IS, establishing discriminant validity, and addressing skepticism with empirical evidence, these challenges have been largely overcome. Although additional challenges face the neuroscientist who wishes to study inspiration, similar challenges have already been overcome in relation to insight and other constructs. We believe that the stage has been set for a rigorous neuroscience of inspiration.

Brain-level explanations of an inspiration episode can then be integrated with explanations at other levels of analysis to produce a richer and more holistic understanding of inspiration. This deeper understanding will aid in determining how and why individuals sometimes feel (or do not feel) compelled to act on their creative ideas. Inspiration has the power to effect change not just for individuals, but also for societies. Technological advancements, cures for diseases, and solutions to environmental problems first emerge as promising ideas. It is difficult to overstate the importance of figuring out why, how, and for whom creative ideas to societal problems fire the soul and inspire the idea actualization process.

## Conflict of interest statement

The authors declare that the research was conducted in the absence of any commercial or financial relationships that could be construed as a potential conflict of interest.

## Footnotes

^1^In these instances, social desirability was assessed using either the Marlowe-Crowne Social Desirability Scale (Crowne and Marlow, 1960) or the Paulhus Deception Scales (Paulhus, 1998).

^2^We note that the Consensual Assessment Technique has also been used to assess the creativity of ideas (e.g., Faure, 2004). Here, we refer specifically to the use of this technique in assessing the creativity of products.

^3^The language of the items and response options of the Inspiration Scale (IS) eliminate this problem by clearly using the term “inspiration” to mean a state, not a cognition or idea.

^4^The authors empirically tested the *transmission model*, which specifies that inspiration mediates the relation between the creativity of the seminal idea and the creativity of the product. Two alternate theoretical models, the *epiphenomenon model* and the *self-perception model*, which suggest that creativity of the idea influences both inspiration and creativity of the product, or that creativity of the idea influences creativity of the product which in turn influences reports of inspiration, respectively, were also tested using structural equation modeling. The authors found support for the transmission model of inspiration over the epiphenomenon and self-perception models.
